# Drivers and patterns of microbial community assembly in a Lyme disease vector

**DOI:** 10.1002/ece3.5361

**Published:** 2019-06-13

**Authors:** Lisa I. Couper, Jessica Y. Kwan, Joyce Ma, Andrea Swei

**Affiliations:** ^1^ Department of Biology Stanford University Stanford California; ^2^ Department of Biology San Francisco State University San Francisco California

**Keywords:** 16s rRNA, community assembly, Lyme disease, microbiome, NexGen sequencing, tick

## Abstract

Vector‐borne diseases constitute a major global health burden and are increasing in geographic range and prevalence. Mounting evidence has demonstrated that the vector microbiome can impact pathogen dynamics, making the microbiome a focal point in vector‐borne disease ecology. However, efforts to generalize preliminary findings across studies and systems and translate these findings into disease control strategies are hindered by a lack of fundamental understanding of the processes shaping the vector microbiome and the interactions therein. Here, we use 16S rRNA sequencing and apply a community ecology framework to analyze microbiome community assembly and interactions in *Ixodes pacificus*, the Lyme disease vector in the western United States. We find that vertical transmission routes drive population‐level patterns in *I. pacificus* microbial diversity and composition, but that microbial function and overall abundance do not vary over time or between clutches. Further, we find that the *I. pacificus* microbiome is not strongly structured based on competition but assembles nonrandomly, potentially due to vector‐specific filtering processes which largely eliminate all but the dominant endosymbiont, *Rickettsia*. At the scale of the individual *I. pacificus*, we find support for a highly limited internal microbial community, and hypothesize that the tick endosymbiont may be the most important component of the vector microbiome in influencing pathogen dynamics.

## 1 INTRODUCTION

Tick‐borne diseases pose a serious and increasing threat to global human and animal health as tick distributions expand and new tick‐borne pathogens are identified (Eisen, Kugeler, Eisen, Beard, & Paddock, 2017; Paddock, Lane, Staples, & Labruna, 2016). Controlling tick‐borne diseases has proved exceedingly difficult given the complex ecology of these pathogens, motivating the need for novel control strategies such as genetic engineering of vectors. Recent evidence suggests there are associations between native vector microbes and pathogens, providing a potentially fruitful avenue for disrupting pathogen transmission (Bonnet, Binetruy, Hernández‐Jarguín, & Duron, 2017; Degnan, Yu, Sisneros, Wing, & Moran, 2009; Dennison, Jupatanakul, & Dimopoulos, 2014; Gall et al., 2016; Hawlena et al., 2013; Jones, Knight, & Martin, 2010; Narasimhan et al., 2014; Ponnusamy et al., 2018). However, harnessing the vector microbiome to this end requires a deeper understanding of how microbes interact within the vector.

It is evident that tick microbes can be maternally inherited (vertical transmission), environmentally acquired through the tick spiracles, mouth, or anal pore (environmental transmission), or obtained from host‐blood feeding (horizontal transmission) (Narasimhan & Fikrig, 2015). The microbial community resulting from these processes consists largely of tick symbionts and guest commensals (Clay & Fuqua, 2010; Greay et al., 2018), which can affect vector fitness (reviewed in Bonnet et al., 2017) and have been associated with variation in vector competence (Budachetri et al., 2018; Civitello, Rynkiewicz, & Clay, 2010; Gall et al., 2016; Narasimhan et al., 2014; Telford, 2009). The latter observation motivated an increased number of studies of the role of ecological and environmental factors in shaping the microbiome and the relationship between microbial symbionts and pathogens (Fryxell & DeBruyn, 2016; Gall, Scoles, Magori, Mason, & Brayton, 2017; Kwan, Griggs, Chicana, Miller, & Swei, 2017; Narasimhan et al., 2014; Swei & Kwan, 2017; Van Treuren et al., 2015; Zolnik, Prill, Falco, Daniels, & Kolokotronis, 2016). These reports implicate a complex suite of factors affecting the tick microbiome including geography, sex, and life stage, and provide opposing evidence about the effect of native tick microbiota on pathogen acquisition, highlighting the inherent complexity of tick microbial ecology and obscuring generalizable patterns.

In particular, studies investigating interactions between and among tick endosymbionts and pathogens have found competitive, facultative, or nonexistent microbial associations driven by a variety of underlying mechanisms (reviewed in de la Fuente et al., 2017). For example, numerous studies have found negative correlations between vertically transmitted endosymbionts and the colonization or transmission of tick‐borne pathogens (Gall et al., 2016; Macaluso, Sonenshine, Ceraul, & Azad, 2002; Narasimhan et al., 2014; Steiner et al., 2008; Telford, 2009). However, the suspected underlying mechanisms have varied in each case, ranging from differences in tick gene expression to microclimate and habitat‐related factors. Other studies have found coinfection rates among various tick‐associated microbes to exceed random expectation (Civitello et al., 2010; Mather, Ribeiro, & Spielman, 1987; Zeidner et al., 2000), suggesting mutual facilitation. Still others have found no difference in the microbiome composition of infected and uninfected ticks, suggesting minimal interaction between the native microbiota and pathogens (Clay et al., 2008; Kwan et al., 2017).

In this study, we attempt to address this complexity by investigating the fundamental processes shaping the larval microbiome of *Ixodes pacificus*, the vector of the Lyme disease pathogen in the western United States. We apply a community ecology framework, using each tick microbiome as a community, to explore the assembly and interactions of tick‐associated microbes. Using larval *I. pacificus*, placed in permeable bags in the field, we confine the inputs to the microbiome to environmental and vertical transmission routes and analyze the effects of these routes on microbial diversity, composition, loads, and function. Our results demonstrate a generally limited *I. pacificus* microbiome that is dominated by the vertically transmitted endosymbiont, *Rickettsia,* with population‐level diversity and composition patterns driven by vertically acquired microbes and, to a lesser degree, environmentally acquired microbes.

## 2 MATERIALS AND METHODS

### 2.1 Sample collection

**Figure 1:**
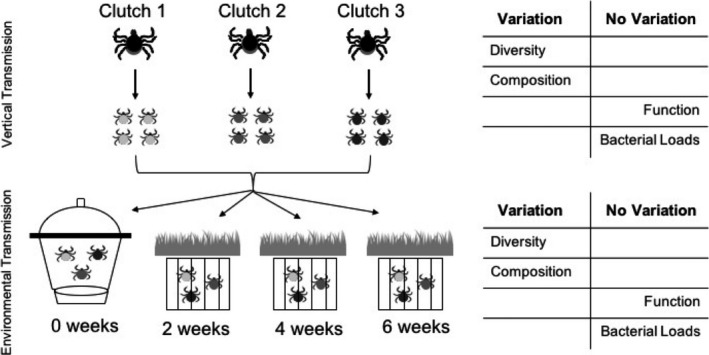
Experimental setup and main findings. Larvae from 3 maternal adults (clutches) were randomly assigned to environmental exposure treatments which included remaining in laboratory, or spending 2, 4, or 6 weeks buried in soil in oak woodland habitat. These vertical and environmental transmission routes generated variation in microbial diversity and composition across clutch and exposure groups, but not in predicted microbial function or microbial loads

Adult female *I. pacificus* were field‐collected from China Camp State Park in Marin County, CA, and fed to repletion on New Zealand white rabbits in the laboratory. Three engorged females were obtained from these means, and collectively produced 93 surviving *I. pacificus* larvae, hatched in the laboratory. These larvae were randomly assigned to spend 0, 2, 4, or 6 weeks in the field environment in an oak woodland forest in northern California (Figure 1). Ticks assigned to the 0‐week group were maintained in the laboratory and, upon hatching, were surface‐sterilized with hydrogen peroxide and ethanol, then flash‐frozen with liquid nitrogen and stored in 70% ethanol. Ticks assigned to all other groups were placed inside sealed but permeable silk, mesh bags following the procedures of Padgett and Lane (2001). Separate mesh bags were used for ticks from each clutch and environmental exposure treatment to ensure we could later identify the treatment group of each tick sample. The silk, mesh bags were hung inside metal cages constructed from wire mesh that was 10 × 10 × 10 cm to prevent predation on ticks from insectivores and birds while still allowing the larvae to move vertically in the soil to avoid desiccation. All cages were buried with the tops of the tick bags exposed to the soil surface. All cages were buried at Pepperwood Preserve in Sonoma County in oak woodland habitat (see Appendix S1 for further details). After 2, 4, or 6 weeks, larvae were gathered, surface‐sterilized, and flash‐frozen on site, then stored in 70% ethanol.

### 2.2 Microbiome sample preparation and sequencing

All ticks were thoroughly surface‐sterilized with successive washes of hydrogen peroxide, ethanol, and de‐ionized H_2_0 to remove environmental contamination (Rudolf et al., 2009). Whole ticks were individually pulverized using a sterilized pestle. Genomic DNA was then extracted using a Qiagen DNeasy Extraction Kit (Qiagen) following the manufacturers’ specifications and using an elution volume of 100 μl. Libraries from individual ticks were then prepared for 16S sequencing following the Illumina MiSeq 16S Metagenomic Sequencing Library Preparation Protocol (Klindworth et al., 2013) with amplicon primers targeting the V3‐V4 hypervariable region. Each sample was amplified in triplicate to reduce PCR bias and then pooled for DNA purification using paramagnetic beads (Appendix S1). To enable differentiation postsequencing, purified amplicons were then barcoded using dual‐index primers with Illumina adapters supplied in a Nextera XT Index Kit (Illumina). A combined library was then prepared by combining equimolar concentrations of all purified, barcoded samples. This combined library contained all 93 larvae, the 3 maternal adults, and 3 negative controls originating from the DNA extraction step (Appendix S1). The library was sequenced on an Illumina MiSeq using the V3 reagent cartridge (300 base pair, paired‐end).

### 2.3 Sequence analysis

Sequence reads were quality‐filtered and processed using QIIME (for more details, see Appendix S1). In total, 84% of reads passed quality filter with an average of 44,995 reads per sample. Sequences were clustered at 97% sequence similarity and rarefied to a depth of 10,182 reads per sample (Appendix Figure S1) to correct for uneven sampling. Rarefying to this depth retained 68 of the original 93 tick samples (see Appendix Table S1 for sample size by treatment). Reads were then assigned to operational taxonomic units (OTUs) using an open reference picking strategy with the NCBI taxonomic database (Benson, Karsch‐Mizrachi, Lipman, Ostell, & Wheeler, 2006). The resulting table, the “normalized OTU table,” contains columns representing tick samples with a standardized number of sequence reads and rows representing OTUs, reported here at the genus level. Operational taxonomic units not accounting for at least 1% of the reads for any sample were pooled into a rare genera category to minimize the impact of sequencing artifacts on diversity calculations (Kunin, Engelbrektson, Ochman, & Hugenholtz, 2010). We further identified and removed OTUs more abundant in negative controls than real samples (Rynkiewicz, Hemmerich, Rusch, Fuqua, & Clay, 2015) using the *decontam* package in R (v3.4.3) (Davis, Proctor, Holmes, Relman, & Callahan, 2018) OTUs flagged as likely contaminants typically included genera such as *Propionibacterium*, which are common human skin microbes, demonstrating that sterile technique during sequencing preparation may not eliminate all contaminant microbes (Glassing, Dowd, Galandiuk, Davis, & Chiodini, 2016). After these quality‐filtering steps, a total of 23 genera, including the rare genera category, were retained for downstream analysis on the remaining 65 larvae and 3 adult samples. While the quality‐filtering steps may have removed or pooled rare microbes with important functions (Jousset et al., 2017), we cannot distinguish these microbes from suspected contaminants, and find it more defensible to pool these genera than to leave in hundreds or thousands of dubious OTUs.

### 2.4 Bacterial load quantification

To measure the overall abundance, or load, of microbiota present in the tick microbiome, we used a SYBR‐based quantitative PCR on the 16S rRNA gene (Appendix S1) (Bacchetti De Gregoris, Aldred, Clare, & Burgess, 2011). Of the original 93 larval samples, only 71 had sufficient volume of DNA extract remaining for testing. The qPCR was performed on these 71 samples which spanned all treatment groups, and each sample was tested in triplicate with each replicate requiring a template volume of 7 μl (Appendix Table S5). Another qPCR protocol targeting the genes encoding outer membrane protein A was employed to quantify the load of the dominant *I. pacificus* endosymbiont, *Rickettsia* phylotype G021 (Appendix S1) (Cheng, Vigil, Schanes, Brown, & Zhong, 2013b). This qPCR was conducted on the 54 samples with remaining DNA extract, and each sample was tested in triplicate with each replicate requiring a template volume of 5 μl. These 54 samples did not include any larvae from clutch 2, as there was no DNA extract remaining from samples in this group. Thus, analysis of *Rickettsia* abundance by clutch pertains only to clutch 1 and clutch 3.

### 2.5 Community ecology analysis

We defined the microbiome of an individual tick as a community and applied standard community ecology analysis techniques to measure microbial presence, abundance, and composition within ticks and compare these metrics across ticks from different clutches (i.e., arising from different adult females) and field exposure times. To characterize microbial alpha diversity within a tick, species richness and evenness were calculated manually using the fully quality‐filtered dataset. Shannon's diversity, which is calculated from both richness and evenness of OTUs, was also calculated for all ticks using the *vegan* package in R (Dixon, 2003). To incorporate phylogenetic differences between species, we also calculated Faith's phylogenetic diversity using the R package *picante* (Kembel et al., 2010) (see Appendix Figure S10 for phylogenetic tree). Diversity values were then log‐transformed to meet the normality assumption of the ANOVA used to detect differences between groups.

To compare tick microbiome composition across treatments, the distance between microbial communities was measured using two metrics targeting different community features (see Appendix S1 for further details). The Jaccard dissimilarity index was used to measure differences in microbial presence–absence, and the Bray–Curtis dissimilarity index was used to capture differences in microbial abundance. These pairwise dissimilarity indices were calculated for all possible pairs of ticks using the *vegdist* function from the *vegan* package in R. We then partitioned these dissimilarity matrices based on clutch and field exposure times through permutational multivariate analysis of variance (PERMANOVA) using the *vegan* function *adonis*, and obtained significance values through permutation of the raw data (*n* = 5,000). To determine where the differences occurred, post hoc tests were conducted using the *pairwise.adonis* function. All *p*‐values were corrected for multiple testing using the Benjamini–Hochberg false discovery rate (FDR) procedure (Benjamini & Hochberg, 1995). To visualize these results, the weighted and unweighted dissimilarity indices were plotted using nonmetric multidimensional scaling (NMDS), an ordination technique which transforms highly dimensional data into a two‐dimensional representation, implemented using the *phyloseq* package in R (McMurdie & Holmes, 2013).

### 2.6 Core microbiome analysis

To determine which OTUs constituted the core *I. pacificus* microbiome, we used indicator species analysis using the *indicspecies* package in R (Cáceres & Legendre, 2009). This analysis distinguishes the genera specific to certain treatment groups (i.e., specific to a certain clutch or exposure time) from those shared among all *I. pacificus* microbiomes. Indicator OTUs were those genera with a statistically significant association (*p* < 0.01, FDR corrected) to a particular clutch or exposure period. *p*‐values were then generated through a permutation test using 1,000 permutations of the original data test, and were corrected for multiple testing using the Benjamini–Hochberg procedure. Core microbiota were defined as those OTUs occurring in all treatments, an association which cannot be statistically tested as there is no external group for comparison (Cáceres, 2013).

### 2.7 Co‐occurrence and network analysis

To test general assembly rules governing the tick microbiome and measure the types of interactions occurring between OTUs therein, we examined the co‐occurrence patterns of the observed OTUs. We calculated checkerboard scores, or C‐scores, which measure the average number of mutual exclusions for each pair of OTUs across a set of communities (Stone & Roberts, 1990). For example, the number of tick samples in which only *Bacillus* or only *Pseudomonas* occur (not both *Bacillus* and *Pseudomonas*) constitutes the number of mutual exclusions, or “checkerboard units,” for this OTU pair. The number of checkerboard units is similarly measured for all possible pairs of OTUs, and averaged to determine the C‐score for the set of communities. These calculations were performed using the *bipartite* package in R (Dormann, Gruber, & Fründ, 2008).

This value was compared to the average C‐score calculated from 5,000 permutations of null communities preserving the marginal totals and connectance from the original data. This approach enabled comparisons of the degree of competitive structuring of the tick microbiome against a null hypothesis of random assortment (Stone & Roberts, 1990). Actual and simulated C‐scores were calculated at the genus level and phylum level to ensure that observed outcomes were not an artifact of the taxonomic rank selected. To further examine microbial interactions, we calculated correlation coefficients for each pair of genera based on their distributions across tick samples. Positively correlated genera were pairs which tended to co‐occur in tick samples, while negatively correlated genera were pairs which co‐occurred less frequently than expected by chance. Significant correlations (*p* < 0.05, FDR corrected) were visualized in a network graph generated using the iGraph package in R (Csárdi & Nepusz, 2006).

### 2.8 Functional role analysis

To estimate the functional role of the microbiota present in *I. pacificus*, the functional gene content was estimated using PICRUSt (v1.1) (Langille et al., 2013). This computational approach uses evolutionary modeling to predict metagenome function from 16S rRNA sequence data and a reference genome database. Briefly, the normalized OTU table was multiplied by the known or inferred gene function for each OTU. Gene functions were then classified into gene family predictions using the KEGG orthology database (Kanehisa, Goto, Sato, Furumichi, & Tanabe, 2012). Differences in gene family predictions between clutches and exposure times were measured via ANOVA using an alpha level of 0.01 and FDR correction. Using PICRUSt, gene predictions can be collapsed into various hierarchical levels; we compared gene predictions between treatment groups at two different hierarchical levels (Level 1 and Level 2) to validate results. The overall feasibility of this approach was assessed by calculating the weighted Nearest Sequenced Taxon Index (NSTI), a measure of the availability of nearby genome representatives for the given OTUs.

## 3 RESULTS

### 3.1 Bacterial load quantification

The absolute abundance of microbes was measured for ticks from all treatment groups via qPCR. Total microbial loads for all groups ranged from 160,438 to 2,182,181 copies/µl, but no significant differences in loads were observed between ticks from different clutches or from different environmental exposure times (Appendix Figure S6). The abundance of *Rickettsia* phylotype G021, the dominant endosymbiont in *I. pacificus*, was also directly measured using a targeted qPCR. Rickettsial loads ranged from 3,052 to 47,987 copies/µl, but again, no significant differences in loads were observed between clutches or between environmental exposure times (Appendix Figure S7).

### 3.2 Community ecology analysis

**Figure 2:**
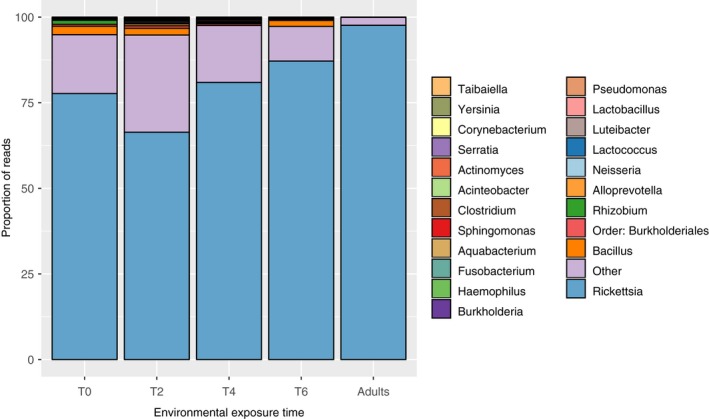
*Ixodes pacificus* microbiome composition after sequence quality filtering. Each of the 5 vertical bars represents an averaged microbiome for samples from that treatment. Each color represents a different OTU present in the microbiome, shown here at the genus level, and the heights of each bar represent the proportion of reads attributed to that OTU. The “Other” category represents all OTUs which did not meet the criteria of presence in at least one sample at ≥1% relative abundance. For similar community composition barplots with genera pooled at a higher level, see Appendix Figure S8

**Figure 3:**
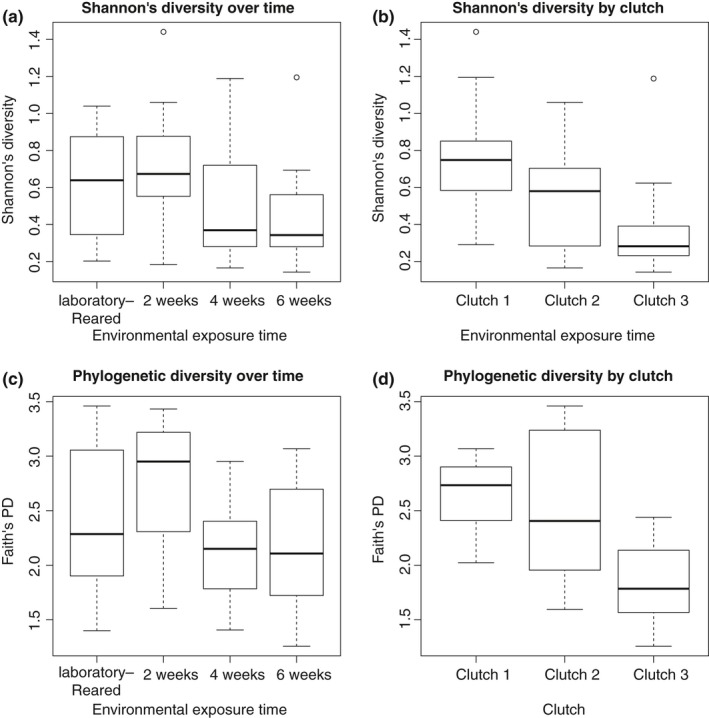
Diversity analysis results. Diversity metrics for larval *Ixodes pacificus* microbiomes based on environmental exposure time (a, c) or clutch (b, d) calculated using either Shannon's diversity index (a, b) or Faith's phylogenetic diversity index (c, d). A single or double star denotes significance at the alpha = 0.05 or 0.01 level, respectively. For raw values, see Appendix Table S4. For OTU richness and evenness plots, see Appendix Figure S3

We obtained quality‐filtered microbiome sequencing results for 65 larvae and 3 adult *I. pacificus* samples. A total of 22 OTUs, excluding the rare genera category, were found across all larval samples, with an average of 6.7 ± 2.4 OTUs per tick (Figure 2 and Appendix Figure S8). In analyzing *I. pacificus* alpha diversity across clutches, we detected significant differences in species richness, species evenness, and Shannon's diversity (ANOVA *F* = 4.0, 9.50, 12.85; *df* = 2; *p* = 0.024, <0.001, <0.001) (Figure 3 and Appendix Figure S3), as well as Faith's phylogenetic diversity (ANOVA *F* = 16.33, *df* = 2; *p* < 0.01) (Figure 3c,d). Comparing across environmental exposure groups, we observed a significant decrease in species evenness, Shannon's diversity, and Faith's phylogenetic diversity over time (ANOVA *F* = 3.32, 3.02, 4.0, *df* = 3, *p* = 0.026, 0.037, 0.012) (Figure 3 and Appendix Figure S3), but no difference in species richness (ANOVA *F* = 1.61; *df* = 3; *p* = 0.20). However, post hoc tests indicated that evenness only differed significantly between the 0‐week and 4‐week treatments; Faith's phylogenetic diversity only differed significantly between the 2‐ and 4‐week treatments and 2‐ and 6‐week treatments; while richness and Shannon's diversity did not differ significantly between any pairs. When grouping all environmentally exposed larvae and comparing these to the laboratory‐maintained larvae, we found significantly lower evenness and Shannon's diversity in environmentally exposed larvae (ANOVA *F* = 7.07, 5.61; *df* = 1; *p* = 0.01, 0.02) (Appendix Figure S2), but no difference in richness or Faith's phylogenetic diversity (ANOVA *F* = 0.14, 0.22, *df* = 1, *p* = 0.72, *p* = 0.63).

**Figure 4:**
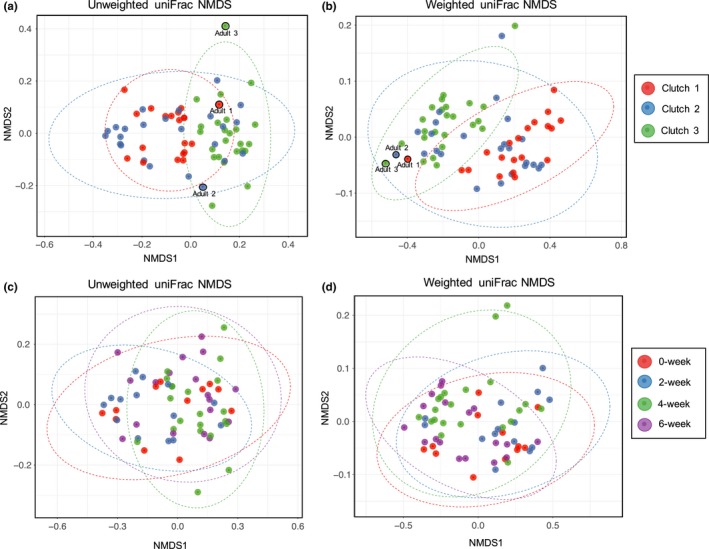
Nonmetric multidimensional scaling (NMDS) of tick microbiomes. (a, b) Unweighted and weighted NMDS for larvae based on clutch. The 3 adults are included to show relatedness between larvae and adult for each clutch. (c, d) Unweighted and weighted NMDS for larvae based on environmental exposure time. Ellipses in all plots represent a 95% confidence interval around the centroid of each group

Analyzing *I. pacificus* microbiome composition revealed further differences between ticks from different clutches and environmental exposure times. Both weighted and unweighted dissimilarity metrics indicated that tick microbiome composition differed significantly by clutch (Bray–Curtis, *F* = 10.89, *df* = 2, *p* < 0.01; Jaccard, *F* = 4.49, *df* = 2, *p* < 0.01). Further, post hoc tests indicated that all clutches were significantly different from each other (*p* < 0.05 for all). Weighted and unweighted dissimilarity metrics also indicated that tick microbiome composition varied significantly with environmental exposure time (Bray–Curtis, *F* = 10.89, *df* = 3, *p* < 0.01; Jaccard, *F* = 2.47, *df* = 3, *p* = 0.024). However, post hoc tests indicated this result was driven by differences in microbiome composition at 2 weeks and 6 weeks (*p* < 0.01); significant differences were not observed for any other pairs. After grouping all environmentally exposed larvae and comparing these to the laboratory‐maintained larvae, no difference in composition was detected. These results were visually supported through the NMDS ordination of the microbiome composition by clutch and time (Figure 4a–d).

### 3.3 Core microbiome analysis

Indicator species analysis was used to identify OTUs associated with ticks from specific clutches or times (indicator species) and those associated with ticks from all treatments (core microbiota). Indicator species included microbes from the genera *Fusobacterium*, *Haemophilus, Lactobacillus, Lactococcus, Neisseria,* and the order *Burkholderiales* (Appendix Table S2). Conversely, microbes within the genera *Bacillus, Clostridium, Rhizobium,* and *Rickettsia* were found to occur across all clutches, and *Clostridium, Lactococcus*, and *Rickettsia* occurred across all field exposure time groups. However, only *Rickettsia* was found in the microbiome of every individual tick and accounted for 78.9% (*SD* = 17.4%) of the sequences obtained across all samples.

### 3.4 Co‐occurrence and network analyses

**Figure 5:**
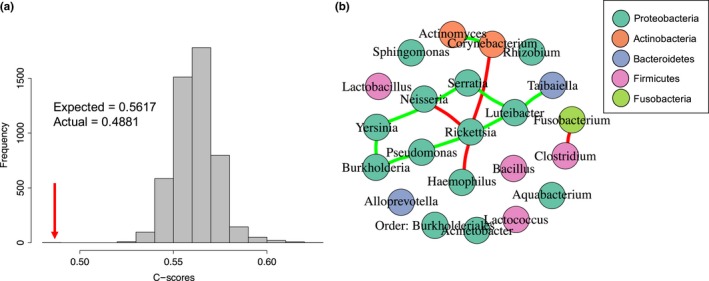
Microbial co‐occurrence analysis patterns. (a) The distribution of C‐scores, a measure of species co‐occurrence, is shown here with OTUs aggregated at the genus level. As indicated by the arrow, the actual C‐score is significantly lower than that of the simulated communities, indicating a nonrandom species assembly process. (b) Network analysis reveals a lack of microbial competition in the *Ixodes pacificus* microbiome. The interaction network shows the pairwise correlations between all OTUs (genera) found across all samples and treatments. Nodes represent genera found in the microbiome and are colored by phylum. Edges represent significant pairwise correlations between genera. Red edges denote negative correlations, and green edges denote positive correlations

Community assembly rules were tested by examining the co‐occurrence patterns of OTUs across tick microbiome samples. The observed checkerboard score, an index measuring the number of species which never co‐occur, was significantly lower than that of the simulated communities, indicating that there were fewer negative interactions among OTUs than expected by chance (Stone & Roberts, 1992). This result was upheld for OTUs aggregated at both the genus and phylum level (*Z* = −6.59, −7.17; *p* < 0.001), enabling us to reject the null hypothesis of neutral assortment (Appendix Figure S4). Similarly, correlation coefficients calculated for each pairwise combination of OTUs revealed fewer significant negative correlations (*n* = 4) than positive correlations (*n* = 7) (Figure 5).

### 3.5 Functional role analysis

PICRUSt, a functional gene content prediction approach, was utilized to estimate the functional role of the *I. pacificus* microbiome based on the 16S rRNA sequencing data. The average weighted Nearest Sequenced Taxon Index (NSTI) was 0.0134 (*SD* = 0.0004), indicating that our samples were highly tractable for metagenome prediction (Appendix S1) (Langille et al., 2013). Comparisons of the predicted gene pathways across clutches and exposure times yielded no significant differences between groups regardless of the hierarchical level at which the gene pathways were collapsed (Appendix Figure S5). That is, the estimated functional gene content of the *I. pacificus* microbiome did not vary by treatment, despite differences in species composition and diversity.

## 4 DISCUSSION

We examined the process of community assembly in the initial, posthatching *I. pacificus* microbiome, and found individual ticks to harbor a depauperate microbial community. Specifically, the average *I. pacificus* microbiome contained only 7 OTUs and was numerically dominated by the endosymbiont, *Rickettsia*. This finding is consistent with recent work combining direct microbial visualization of *I. pacificus* with sequencing methods (Ross et al., 2018), but contradicts previous reports indicating that Ixodid ticks harbor highly diverse microbiomes, including hundreds to thousands of OTUs (Andreotti et al., 2011; Budachetri et al., 2014; Carpi et al., 2011; Estrada‐Peña, Cabezas‐Cruz, Pollet, Vayssier‐Taussat, & Cosson, 2018; Nakao et al., 2013; Ponnusamy et al., 2014; Fryxell & DeBruyn, 2016; Rynkiewicz et al., 2015, Zhang et al., 2014; Zolnik et al., 2016). While variation in microbial richness observed across studies may be due to differences in the precise species or life stages examined (Kwan et al., 2017; Van Treuren et al., 2015), the difference in OTU richness in our study and those aforementioned is orders of magnitude greater than that typically generated by species‐ and life stage‐specific differences. Evidence suggests that the high diversity estimates previously reported are inflated due to contamination (Salter et al., 2014; Weiss et al., 2014), sequencing error (Huse, Welch, Morrison, & Sogin, 2010), or the presence of transient microbes (Wang, Gilbreath, Kukutla, Yan, & Xu, 2011; Zolnik, Falco, Daniels, & Kolokotronis, 2018). While the raw sequence data obtained in this study similarly suggested the presence of thousands of OTUs, we used rigorous statistical methods to control for potential contamination and sequencing error. Further, we used high sample replication and sequencing results from multiple time points to better differentiate rare or transient microbes from common tick microbial residents. Using these methods, we found strong support for a highly limited internal *I. pacificus* microbiome.

Despite a limited microbiome within individual ticks, drivers and patterns of microbial diversity were discernible at the population level. Specifically, we found that maternal identity and environmental exposure period generated significant variation in microbiome diversity and composition. However, these vertical and environmental transmission routes did not generate differences in overall microbial abundance, endosymbiont abundance, or functional gene content. These results indicate that microbial diversity and composition patterns do not necessarily reflect differences in microbial function and that abundance and function appear to be more highly conserved features of the *I. pacificus* microbiome.

We further found that while ticks can acquire internal microbiota from their environment, microbiome diversity decreased with increasing environmental exposure time. Additionally, microbial diversity was lower in environmentally exposed larvae compared to laboratory‐maintained larvae, but composition did not differ between these groups. These results suggest that environmental transmission plays a relatively fleeting role in shaping the microbiome, in contrast to prior findings that environmentally acquired microbes contribute substantially to the tick microbiome (Carpi et al., 2011; Greay et al., 2018; Kwan et al., 2017; Zolnik et al., 2016).

To investigate the processes underlying the observed reduction in microbial diversity over time, we examined microbial interactions through co‐occurrence analysis. We found fewer negative interactions than expected by chance, suggesting that the *I. pacificus* microbiome is not strongly shaped by microbial competition. Specifically, we found fewer microbial pairs with nonoverlapping distributions in the true community than in a null community model, and fewer negative pairwise correlations than positive correlations. These results suggest that the observed decline in *I. pacificus* microbial alpha diversity over time was not driven by competition among microbes but were potentially imposed by environmental conditions within the tick itself (Costello, Stagaman, Dethlefsen, Bohannan, & Relman, 2012; Faust et al., 2012). The corresponding decrease in phylogenetic diversity further suggests that conditions within the tick may shift over time allowing only certain more closely related taxa to persist in the environment (Webb, 2000; Webb, Ackerly, McPeek, & Donoghue, 2002). Environmental filtering, in which habitat conditions allow only taxa with particular traits or phenotypes to persist, is a common phenomenon in bacterial communities (Horner‐Devine & Bohannan, 2006; Newton, Jones, Helmus, & McMahon, 2007). While the process of environmental filtering was not directly explored here, evidence of the host niche dictating microbial assembly, similar to that seen here, has previously been observed across diverse study systems including *C. elegans* (Berg et al., 2016), fish (Schmidt, Smith, Melvin, & Amaral‐Zettler, 2015), cacti (Fonseca‐García et al., 2016), and humans (Levy & Borenstein, 2013). In ticks, the microbial selection process may be driven by internal tick characteristics such as redox conditions and pH (Hyde, Trzeciakowski, & Skare, 2007; Narasimhan et al., 2014), low quantities of essential nutrients (Narasimhan & Fikrig, 2015), innate immune response used to control microbes (Hajdušek et al., 2013; Sonenshine & Roe, 2013), or microbe–microbe competition (Devevey, Dang, Graves, Murray, & Brisson, 2015; Gall et al., 2016). As tick microbes vary greatly in their contribution to vector fitness, we hypothesize that *I. pacificus* creates favorable conditions for microbes providing strong fitness benefits, perhaps at the expense of other microbes (Burgdorfer, 1988; Macaluso et al., 2002).

Identifying the core microbiome, those microbes present within all or the vast majority of assemblages (Turnbaugh & Gordon, 2009), allowed for further exploration of tick–microbe associations. Although 22 OTUs were found across tick samples after rigorous sequence quality control measures, only *Clostridium* and *Rickettsia* were consistently found in ticks across clutches and time points (Appendix Table S3). The role of *Clostridium* within the tick remains unknown, but this common environmental bacterium has been reported in various Ixodid tick species around the world (Andreotti et al., 2011; Carpi et al., 2011; Clow, Weese, Rousseau, & Jardine, 2018). Further, *Clostridium* was detected in larvae not exposed to the environment, suggesting that this microbe can also be acquired through vertical transmission. However, *Clostridium* was only found in 55% of ticks sampled, while *Rickettsia*, specifically *Rickettsia* phylotype G021, was present in every sample, and has been found in 100% of *I. pacificus* sampled by separate studies (Cheng, Lane, Moore, & Zhong, 2013a; Kwan et al., 2017). Prior reports have found this microbe to be vertically and transstadially transmitted at 100% efficiency, to increase significantly after engorgement, and to play a nutritional role within ticks (Cheng, Vigil, et al., 2013b). Specifically, this *Rickettsia* phylotype has the demonstrated capability of synthesizing folate (Hunter et al., 2015), an essential vitamin absent from the ticks’ blood‐based diet and which ticks lack the genetic capacity to synthesize de novo (Hill & Wikel, 2005; Pagel Van Zee et al., 2007). These reports provide strong evidence that *Rickettsia* plays a beneficial, potentially essential, role in *I. pacificus* development, thus incentivizing its proliferation within the tick in a manner that may be detrimental to other bacteria.

*Rickettsia* may not be the only beneficial microbe, as the role of the other detected genera within the tick is largely unknown, but its high proportion in the sequence reads and the paucity of other genera represented broadly across samples further indicates that unfed, larval *I. pacificus* harbor a low‐diversity internal microbiome. The presence of a depauperate microbiome, and the dominance of specific, beneficial microbes, highlights the need to determine the role of tick endosymbionts in pathogen transmission. While other environmentally acquired microbes such as *Bacillus, Pseudomonas,* and *Enterobacteriaceae* may limit pathogen colonization in *Ixodes* (Ross et al., 2018), these microbes are not reliably vertically transmitted, and their ability to persist in the tick during a molting event is unknown. Endosymbionts such as *Rickettsia,* however, are efficiently vertically transmitted and have been associated with reductions in pathogen acquisition in some tick‐borne pathogen systems (Gall et al., 2016; Macaluso et al., 2002; Telford, 2009). However, the mechanisms by which *Rickettsia*, an intracellular microbe (Winkler, 1990), interact with pathogens remain unknown, and associations between *Rickettsia* and pathogen presence or loads are not consistently found (Kwan et al., 2017). Our results found that, while negative microbial interactions were relatively rare within the *I. pacificus* microbiome, *Rickettsia* was involved in the majority of the negative interactions. It remains to be determined whether *Rickettsia* is directly outcompeting these other microbes, or opportunistically or independently increasing as others decrease within the tick over time. Elucidating the role of *Rickettsia*, and tick endosymbionts generally, in tick‐borne pathogen dynamics, constitutes a critical next step forward in this field.

Overall, we demonstrate the use of a community ecology framework for investigating features of the vector microbiome. Characterizing the initial microbial community assembly patterns using thoroughly filtered sequence data to avoid spurious conclusions provided a framework for evaluating vector microbial variation and interactions. In this study, we demonstrate that vertical transmission routes contribute to population‐level patterns of microbial diversity and composition within *I. pacificus*, with environmental transmission playing a minimal role. Despite discernible patterns at the population level, we found that individual ticks harbor low‐diversity microbiomes, increasingly dominated by the vertically transmitted endosymbiont, *Rickettsia*. Given the dominance of this endosymbiont as well as tick‐associated endosymbionts in general (Narasimhan & Fikrig, 2015), future efforts to investigate tick microbiomes for applications in disease prevention may want to focus on the role of vector‐associated endosymbionts in pathogen acquisition, maintenance, and transmission. In particular, investigating how vector competence varies with obligate endosymbiont burdens or facultative endosymbiont presence may identify microbes capable of disrupting pathogen transmission that may be harnessed in novel strategies to prevent tick‐borne disease.

## CONFLICT OF INTEREST

None declared.

## AUTHOR CONTRIBUTIONS

J.Y.K. and A.S. designed the project; J.Y.K and J.M performed the field collections; J.Y.K, J.M, and L.I.C performed the laboratory research; A.S. provided new reagents and analytical tools; L.C analyzed the data; and L.C. and A.S. wrote the manuscript.

## Data Availability

The project OTU table is available on Dryad with DOI accession number: 10.5061/dryad.2nv32qh.
