# Supplementary material for: Drivers and patterns of microbial community assembly in a Lyme disease vector
Source: Ecol Evol. 2019 Jun 13;9(13):7768–79. doi: 10.1002/ece3.5361 (PMC6635933; doi:10.1002/ece3.5361)
Supplement: Supplementary file 1 [file ECE3-9-7768-s001.docx]

**Appendix**

**Methods:** *Field manipulation*

Ticks were placed inside of 7x10cm silk screen packets according to treatment group. Packets were buried vertically in the soil inside one of three 10x10x10cm wire mesh cages. Mesh bags were randomly assigned to cages such that a variety of treatment groups were held within each cage. Cages were covered with leaf litter and soil, and the tops of each packet were exposed to the soil surface. Larvae were left in mesh bags for 2, 4, or 6 weeks to reflect meaningful development periods for *I. pacificus* larvae as well as to maximize field survivorship. *I. pacificus* eggs are typically laid February – May (Padgett & Lane, 2001), and larval host-seeking typically occurs between late April – June (Eisen, 2001). Thus 2-week intervals likely span development periods of *I. pacificus*, potentially resulting in variation in the tick microbiome which we were intending to capture here.

**Methods:** *Microbiome sampling preparation*

After genomic DNA extraction, individual ticks were subjected to amplicon PCR targeting the V3-V4 hypervariable region of 16S rRNA using the primer sequences listed in Klindworth *et al.* (2013) and cycling conditions: 95 °C for 3 minutes; 25 cycles of: 95 °C for 30 s, 57 °C for 30 s, 72 °C for 30 s; and a final extension at 72 °C for 5 min. To increase yield and decrease PCR bias, each sample was amplified in triplicate and pooled for DNA purification (Polz & Cavanaugh, 1998). To remove non-specific banding resulting from the amplicon PCR, we performed DNA purification using paramagnetic beads as described in Couper *et al.,* 2018. Individual tick samples and negative controls were then barcoded using index PCR primers from a Nextera XT Index Kit (Illumina, San Diego, CA, USA) and cycling conditions: 95 °C for 3 min; 8-14 cycles of: 95 °C for 30 s, 55 °C for 30 s, 72 °C for 30 s; and a final extension at 72 °C for 5 min. Samples were amplified in duplicate and pooled for DNA purification using paramagnetic beads. To obtain equimolar sample concentrations prior to loading, each sample was subject to library quantification via qPCR with a KAPA Library Quantification Kit (KAPA Biosystems, Wilmington, MA, USA). The qPCR was performed in a 10 L reaction containing 6.0 L of qPCR master mix (from library quantification kit), 2.0 L ddH_2_O, and 2.0 L sample or standard. Samples and standards were run in triplicate and at three dilution levels to ensure accurate quantification. The qPCR was performed as specified in the KAPA library quantification kit protocol. All samples were then diluted to 4 nM and combined in equal volumes to create the final combined library. The combined library was then quantified using the same methods as above to confirm the 4 nM concentration. The final library was then denatured and diluted to 12 pM for loading on an Illumina MiSeq using V3 reagent cartridge along with a 25% PhiX sequence control spike-in to increase library diversity.

**Methods:** *Sequence Quality Filtering*

The sequencing quality filtering pipeline consisted of 1) Illumina internal quality filtering, 2) rarefaction to correct for uneven sequencing reads between samples, 3) pooling of rare genera to minimize effects of contamination and sequencing error, and 4) decontamination to further identify likely contaminant microbes.

*1) Illumina quality filter.* A total of 12,885,713 paired-end reads passed the Illumina internal quality filter process, which removes clusters of reads with less reliable base calls.

*2) Rarefaction.* Rarefaction curves, which relate sequencing effort to OTU discovery, indicated that a sequencing depth of 10,000 reads or greater should allow for accurate quantification of OTU richness across samples (Supplementary Figure S1). Given this, we rarefied to a depth of 10,129 reads per sample as this was the sequence count of the first tick sample with over 10,000 reads. Rarefying samples to this depth preserved 65 larvae, the 3 adult samples, and 2 extract negatives. Across these samples, 1,714 OTUs were identified with an average of 93.3 ± 4.3 OTUs per sample.

*3) Pooling rare genera.* To reduce the impact of possible sequencing error on downstream diversity metrics, all OTUs not accounting for at least 1% of the reads for any sample were pooled (Gall *et al.,* 2016; Kwan *et al.,* 2016) leaving 26 OTUs and a rare genera category (Supplementary Table S1).

*4) Decontamination.* To identify and remove suspected contaminant OTUs, we used the *decontam* package, implemented in R (V3.4.3) (Davis *et al.,* 2017). A threshold of 0.5 was selected for analysis, resulting in any OTU which was more abundant in negative controls than true samples being labeled as a contaminant. This process preserved 22 OTUs and the rare genera category. To further identify suspected contaminant OTUs, we performed linear discriminant analysis effect size implemented through bioBakery, a meta’omic sequence environment (Segata *et al.,* 2011). Samples were grouped as negative controls or real samples for analysis. An alpha value of 0.05 for used for the factorial Kruskal-Wallis test among classes and the pairwise Wilcoxon text between subclasses, and a threshold of 2.0 was used for the logarithmic LDA scores for discriminative features. This process did not identify any additional suspected contaminants.

A total of 23 genera, including the rare genera category, remained after these quality filtering steps.

**Methods:** *Community Ecology Analysis*

We compared microbial composition between samples using two dissimilarity indices. The Jaccard dissimilarity index was used to compare differences in the identity of microbes present between samples, while the Bray-Curtis dissimilarity index was used to compare both microbial identity and relative abundance. These dissimilarity indices were calculated for all possible pairs of tick samples (i.e. dissimilarity between sample 1 and 2, sample 1 & 3, sample 1 & 4, etc.). For a given pair, the indices are calculated as follows:

Jaccard: $1-\frac{a}{(a+b+c)}$

Bray-Curtis: $1- \frac{2C_{ij}}{(S_{i} + S_{j})}$

where *a* indicates the number of OTUs shared between samples, *b* the number of unique OTUs in sample *i*, *c* the number of unique OTUs in sample *j*, $C_{ij}$ the summed abundance of OTUs shared between samples *i* and *j*, $S_{i}$ the total abundance of OTUs in sample *i*, and$S_{j}$ the total abundance of OTUs in sample *j*.

These dissimilarity values, either Jaccard or Bray-Curtis, were calculated for all possible pairs of tick samples. The dissimilarity values for pairs from different treatments (i.e. different clutches or different exposure periods) were then compared with a distribution of dissimilarity values generated through random permutation from the same data set (PERMANOVA).

**Methods:** *Quantitative PCR*

Overall microbial loads were quantified for all ticks with sufficient sample volume remaining after microbiome sequencing, which included 71 larvae across all treatments. Microbial loads were measured using the SYBR-based qPCR protocol targeting the 16S rRNA gene as outlined in Gregoris *et al.,* 2011. All samples were run in triplicate with the following cycling conditions: 95 °C for 5 min; 30 cycles of: 95 °C for 15 s, 61.5 °C for 15 s, 72 °C for 20 s; and a final elongation at 72 °C for 5 min. Endosymbiont loads were also measured for the remaining 54 larvae spanning all treatment groups except clutch 2 which had no larvae with available product remaining. Loads were measured using a TaqMan probe-based assay as outlined in Cheng *et al.,* 2013. This qPCR targets genes encoding the outer membrane protein A, and the specific probe used here targets *Rickettsia* phylotype G021, the dominant endosymbiont of *Ixodes pacificus* (Hunter *et al.,* 2015). We followed the protocol as provided with the modification that we used the primer and probes listed for phylotype G022 to correctly capture the intended phylotype (J. Zhong, personal communication, May 8, 2017). Samples were run in triplicate using the following cycling condition: 94 °C for 10 min; and 40 cycles of: 94 °C for 30 s, 60 °C for 60s.

**Methods:** *References*

Bacchetti De Gregoris, T., Aldred N., Clare A.S., Burgess J.G. (2011). Improvement of phylum- and class-specific primers for real-time PCR quantification of bacterial taxa. *J Microbiol Methods* **86**: 351–356.

Cheng D., Vigil K., Schanes P., Brown R.N., Zhong J. (2013). Prevalence and burden of two rickettsial phylotypes (G021 and G022) in Ixodes pacificus from California by real-time quantitative PCR. *Ticks Tick-Borne Dis* **4:** 280–287.

Couper L.I., Swei A. In press. Tick microbiome characterization by next-generation 16S rRNA amplicon sequencing. *JoVE*.

Davis, N.M., Proctor, D., Holmes, S. P., Relman, D. A., & Callahan, B. J. (2018). Simple statistical identification and removal of contaminant sequences in marker-gene and metagenomics data. Microbiome. **6**: 226. https://doi.org/10.1101/221499

Eisen, R.J., L. Eisen, and R.S. Lane. 2001. Prevalence and abundance of Ixodes pacificus immatures (Acari: Ixodidae) infesting western fence lizards (Sceloporus occidentalis) in northern California: Temporal trends and environmental correlates. J. Parasitol. 87: 1301-1307.

Hunter D.J., Torkelson J.L., Bodnar J., Mortazavi B., Laurent T., Deason J. et al. (2015). The Rickettsia endosymbiont of Ixodes pacificus contains all the genes of de novo folate biosynthesis. *PLoS One* **10**: e0144552.

Klindworth A., Pruesse E., Schweer T., Peplies J., Quast C., Horn M., et al. (2013). Evaluation of general 16S ribosomal RNA gene PCR primers for classical and next-generation sequencing-based diversity studies. *Nucleic Acids Res* **41:** e1.

Padgett, K. A., & Lane, R. S. (2001). Life Cycle of Ixodes pacificus (Acari: Ixodidae): Timing of Developmental Processes Under Field and Laboratory Conditions. Journal of Medical Entomology, 38(5), 684–693. https://doi.org/10.1603/0022-2585-38.5.684.

Polz M.F., Cavanaugh C.M. (1998). Bias in template-to product ratios in multitemplate PCR. Appl *Environ Microbiol* **64**: 3724–3730.

Segata N., Izard J., Waldron L., Gevers D., Miropolsky L., Garrett W.S., et al. (2011). Metagenomic biomarker discovery and explanation. *Genome Bio*l **12**: R60

TaKara Bio. (2018). *Library Quantification Kit: User Manual.* Mountain View, CA.

**Table S1: Sample size by treatment.**

This table lists the tick sample size for each experimental group. Sample numbers indicate the number of samples retained after rarefying to a depth of 10,129 reads as all analyses presented were performed on these samples only.

|  | Clutch 1 | Clutch 2 | Clutch 3 |
| --- | --- | --- | --- |
| 0 weeks | 1 | 6 | 5 |
| 2 weeks | 5 | 7 | 2 |
| 4 weeks | 6 | 7 | 8 |
| 6 weeks | 7 | 3 | 8 |
| Adult | 1 | 1 | 1 |
| **Total** | 20 | 24 | 24 |

**Table S2.** **Indicator Species Analysis.**

This table lists the association between OTUs and specific clutch and environmental exposure treatment groups. The indicator values denote the strength of the association between an OTU and a treatment group, while the FDR-corrected p-values indicates the statistical significance of the association.

|  | **Environmental Exposure Time** | | |
| --- | --- | --- | --- |
|  | Associated Times | Indicator Value | p-value (FDR corrected) |
| Acinteobacter | T4 | 0.431 | 0.43 |
| Actinomyces | T6 | 0.236 | 0.664 |
| Alloprevotella | T2 | 0.523 | 0.171 |
| Aquabacterium | T0, T4, T6 | 0.573 | 0.664 |
| Bacillus | T0, T2, T6 | 0.838 | 0.152 |
| Burkholderia | T2 | 0.459 | 0.213 |
| Clostridium | All | 0.713 | NA |
| Corynebacterium | T0, T2, T6 | 0.423 | 0.49 |
| Fusobacterium | T0, T2 | 0.675 | 0.019 |
| Haemophilus | T0, T2 | 0.588 | 0.028 |
| Lactobacillus | T0, T2, T4 | 0.556 | 0.664 |
| Lactococcus | All | 0.511 | NA |
| Luteibacter | T2, T4 | 0.58 | 0.213 |
| Neisseria | T0, T2 | 0.611 | 0.095 |
| Order: Burkholderiales | T0, T2, T4 | 0.802 | 0.171 |
| Other | All | 1 | NA |
| Pseudomonas | T2, T4, T6 | 0.665 | 0.183 |
| Rhizobium | T2, T6 | 0.809 | 0.1 |
| Rickettsia | All | 1 | NA |
| Serratia | T2 | 0.453 | 0.173 |
| Sphingomonas | T0, T4, T6 | 0.719 | 0.173 |
| Taibaiella | T4 | 0.309 | 0.339 |
| Yersinia | T2 | 0.378 | 0.173 |

|  | **Clutch** | | |
| --- | --- | --- | --- |
|  | Associated Clutches | Indicator Value | P-value (FDR corrected) |
| Acinteobacter | C3 | 0.472 | 0.153 |
| Actinomyces | C1 | 0.224 | 0.32 |
| Alloprevotella | C1, C2 | 0.428 | 0.387 |
| Aquabacterium | C3 | 0.593 | 0.314 |
| Bacillus | All | 0.813 | NA |
| Burkholderia | C1 | 0.443 | 0.175 |
| Clostridium | All | 0.713 | NA |
| Corynebacterium | C1, C2 | 0.457 | 0.175 |
| Fusobacterium | C1, C2 | 0.57 | 0.048 |
| Haemophilus | C2 | 0.626 | 0.009 |
| Lactobacillus | C1 | 0.693 | 0.025 |
| Lactococcus | C1 | 0.69 | 0.022 |
| Luteibacter | C1, C3 | 0.552 | 0.248 |
| Neisseria | C2 | 0.643 | 0.012 |
| Order: Burkholderiales | C1 | 0.96 | 0.009 |
| Other | All | 1 | NA |
| Pseudomonas | C1, C3 | 0.673 | 0.105 |
| Rhizobium | All | 0.784 | NA |
| Rickettsia | All | 1 | NA |
| Serratia | C1, C2 | 0.335 | 0.619 |
| Sphingomonas | C1, C3 | 0.697 | 0.175 |
| Taibaiella | C3 | 0.302 | 0.27 |
| Yersinia | C1 | 0.316 | 0.175 |

**Table S3. OTU presence in samples.**

This table lists the number of individual samples, out of 65, containing each OTU.

| **OTU** | **Number of Samples** |
| --- | --- |
| Acinteobacter | 8 |
| Actinomyces | 1 |
| Alloprevotella | 9 |
| Aquabacterium | 19 |
| Bacillus | 43 |
| Burkholderia | 7 |
| Clostridium | 33 |
| Corynebacterium | 9 |
| Fusobacterium | 15 |
| Haemophilus | 9 |
| Lactobacillus | 18 |
| Lactococcus | 17 |
| Luteibacter | 16 |
| Neisseria | 16 |
| Order: Burkholderiales | 41 |
| Other | 65 |
| Pseudomonas | 26 |
| Rhizobium | 40 |
| Rickettsia | 65 |
| Serratia | 6 |
| Sphingomonas | 31 |
| Taibaiella | 2 |
| Yersinia | 2 |

**Table S4. Diversity metrics.**

This table lists raw values for species richness, evenness, and diversity for each treatment group.

|  | **Species Richness (Mean** ± **SE)** | **Species Evenness (Mean** ± **SE)** | **Shannon's Diversity (Mean** ± **SE)** |
| --- | --- | --- | --- |
| **Clutch 1 Larvae** | 8.53 ± 0.49 | 0.36 ± 0.03 | 0.76 ± 0.06 |
| **Clutch 2 Larvae** | 8.00 ± 0.44 | 0.27 ± 0.02 | 0.57 ± 0.06 |
| **Clutch 3 Larvae** | 6.61 ± 0.54 | 0.20 ± 0.03 | 0.35 ± 0.05 |
| **Time 0 Larvae** | 7.42 ± 0.61 | 0.26 ± 0.03 | 0.50 ± 0.07 |
| **Time 2 Larvae** | 9.14 ± 0.69 | 0.33 ± 0.03 | 0.73 ± 0.09 |
| **Time 4 Larvae** | 7.29 ± 0.44 | 0.26 ± 0.03 | 0.53 ± 0.06 |
| **Time 6 Larvae** | 7.11 ± 0.61 | 0.24 ± 0.03 | 0.43 ± 0.06 |

**Table S5: qPCR sample size by treatment.**

This table lists the tick sample size from each experimental group for which qPCR was performed.

|  | Clutch 1 | Clutch 2 | Clutch 3 |
| --- | --- | --- | --- |
| 0 weeks | 2 | 4 | 7 |
| 2 weeks | 7 | 3 | 7 |
| 4 weeks | 8 | 7 | 4 |
| 6 weeks | 8 | 8 | 6 |
| **Total** | 25 | 22 | 24 |

**Table S5. Suspected contaminant OTUs.**

This table lists taxa more commonly present in negative controls than in true samples, and provides the average relative abundance for each group from most to least abundant in the negative controls.

| **Taxonomy** | **Avg. in Negative Control** | **Avg. in Samples** |
| --- | --- | --- |
| Genus: Delftia | 0.253405 | 0.008866 |
| Genus: Cutibacterium | 0.15876 | 0.043597 |
| Genus: Staphylococcus | 0.038985 | 0.005572 |
| Genus: Delftia | 0.033349 | 0.000971 |
| Family: Xanthomonadaceae | 0.033114 | 0.00158 |
| Genus: Ochrobactrum | 0.025834 | 0.001009 |
| Genus: Pseudomonas | 0.020902 | 0.002002 |
| Genus: Staphylococcus | 0.020432 | 0.001489 |
| Genus: Delftia | 0.018788 | 0.000683 |
| Genus: Pseudomonas | 0.016205 | 0.006266 |
| Genus: Coryneba cterium | 0.015735 | 0.005311 |
| Genus: Propionibacterium | 0.014561 | 0.003928 |
| Genus: Micrococcus | 0.012447 | 0.001687 |
| Genus: Staphylococcus | 0.012447 | 0.001804 |
| Genus: Staphylococcus | 0.011977 | 0.00142 |
| Genus: Pseudomonas | 0.011273 | 0.00024 |
| Genus: Streptococcus | 0.010803 | 0.005188 |
| Genus: Pseudomonas | 0.010333 | 0.000075 |
| Genus: Streptococcus | 0.007985 | 0.000496 |
| Genus: Pseudomonas | 0.007985 | 0.001254 |
| Genus: Stenotrophomonas | 0.007515 | 0.000262 |
| Family: Oxalobacteraceae | 0.007515 | 0.00032 |
| Genus: Haemophilus | 0.00728 | 0.000961 |
| Family: Comamonadaceae | 0.006341 | 0.000016 |
| Genus: Corynebacterium | 0.005871 | 0.002562 |
| Genus: Propionibacterium | 0.005167 | 0.001559 |
| Genus: Herbaspirillum | 0.005167 | 0.000475 |
| Genus: Corynebacterium | 0.004932 | 0.002797 |
| Genus: Porphyromonas | 0.004697 | 0.000534 |
| Genus: Lactobacillus | 0.004462 | 0.000886 |
| Family: Neisseriaceae | 0.001644 | 0.001452 |
| Genus: Corynebacterium | 0.001409 | 0.000358 |
| Genus: Corynebacterium | 0.001409 | 0.00047 |
| Genus: Granulicatella | 0.001174 | 0.000667 |
| Genus: Aquabacterium | 0.00047 | 0.000342 |

**
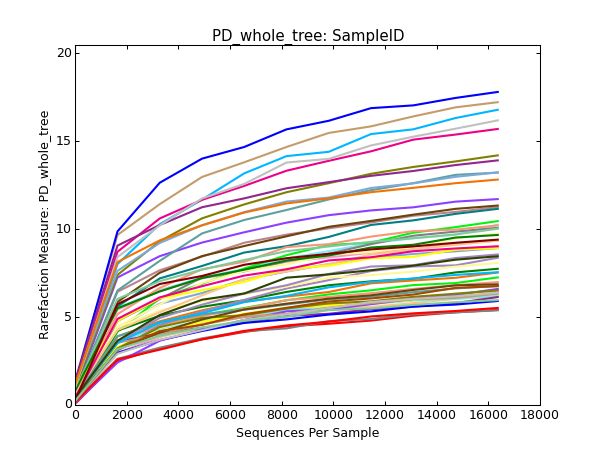
**

**Supplementary Figure S1:** This rarefaction curve displays the relationship between sequencing depth and the discovery of observed operational taxonomic units (OTUs). Each line represents OTU accumulation for an individual tick sample. A sequencing depth of 10,129 reads was selected in this study as the curve appears to level off after 10,000 reads.

**Supplementary Figure S2.** Microbiome species richness, evenness, and Shannon’s diversity are depicted here aggregated by whether larvae were lab-maintained (“Lab”), or environmentally-exposed (“Field”). Lab-maintained larvae were those exposed to the environment for 0 weeks, while field larvae were exposed for 2, 4, or 6 weeks. The horizontal bar and star denote significance at the alpha = 0.05 level.

**a**

**b**

**c**

**d**

**Supplementary Figure S3.** Microbiome species richness and evenness based on clutch **(a-b)**, and environmental exposure time **(c-d)**.

**Supplementary Figure S4:** The distribution of C-scores, a measure of species co-occurrence, is shown here with OTUs aggregated at the **(a)** genus level and **(b)** phylum level. In both instances, the observed C-score is significantly lower than that of the simulated communities, indicating a non-random species assembly process.

**Supplementary Figure S5:** Predicted gene functions obtained from PICRUSt are depicted here for samples aggregated by **(a)** clutch and **(b)** environmental exposure time. Functional pathways are collapsed at the third hierarchical level available, and include: cellular processes, environmental information processing, genetic information processing, human diseases, metabolism, organismal systems, and a classified grouping. Error bars represent one standard deviation from the mean.

**Supplementary Figure S6.** Total bacterial loads, as obtained via qPCR of the 16s rRNA gene, are shown here for larvae aggregated by clutch and by environmental exposure time. No significant differences in total loads were detected between any treatment groups.

**Supplementary Figure S7.** *Rickettsia* loads, as obtained via qPCR of the ompA gene, are shown here for larvae aggregated by clutch and by time. No significant differences in *Rickettsia* loads were detected between any treatment groups.

**Supplementary Figure S8.** *Ixodes pacificus* microbiome composition after sequence quality filtering. Each of the 5 vertical bars represent an averaged microbiome for samples from that treatment. Each color represents an OTU or group of OTUs. The ‘Rare Genera’ category includes all those OTUs which did not meet the criteria of presence in at least one sample at $\geq$1% relative abundance. The ‘Other’ category contains all remaining 21 OTUs, combined here for easier viewing. The heights of each bar represent the proportion of reads attributed to that category. For similar community composition barplots with colors for each genera separately, see Figure 2.

**Supplementary Figure S9. (a)** Weighted and (b) unweighted non-metric multidimensional scaling (NMDS) of tick microbiomes based on whether ticks were exposed to the field environment or maintained in the lab. Ellipses in all plots represent a 95% confidence interval around the centroid of each group.

**Supplementary Figure S10.** Phylogenetic tree containing microbes identified through sequencing of all larval *Ixodes pacificus* samples and maintained after the sequencing quality filtering pipeline. Taxonomic information is displayed at the genus level unless otherwise specified.
